# Mental and physical conditions associated with physical inactivity among Farhangian University students during virtual classes: A cross-sectional study

**DOI:** 10.3389/fpsyg.2023.1094683

**Published:** 2023-03-13

**Authors:** Morteza Homayounnia Firouzjah, Morteza Pourazar, Saeed Nazari Kakvandi

**Affiliations:** ^1^Department of Physical Education, Farhangian University, Tehran, Iran; ^2^Faculty of Sport Sciences, Ferdowsi University of Mashhad, Mashhad, Iran

**Keywords:** activity, Farhangian University, virtual classes, mental complications, student

## Abstract

**Background:**

The level of mobility and general health has decreased among students in virtual classes during COVID-19 pandemic. The present cross-sectional study aims to investigate the mental and physical conditions related to inactivity among the students of Farhangian University during the virtual classes.

**Methods:**

This is a cross-sectional study. 475 students (214 females and 261 males) were selected as the statistical sample of the study based on Morgan’s Table from Farhangian University, Iran. The statistical population includes students studying at Farhangian University of Mazandaran province that using Convenience Sampling the sample size based on Morgan’s Table, 475 students consisting of 214 females and 261 males were randomly selected as the statistical sample of the study. The research instruments of this study include International Physical Activity Questionnaire, Saehan Caliper (SH5020), Coopersmith Self-Esteem Scale, Beck Depression Questionnaire, and Nordic Skeletal and Muscular Disorders Questionnaire. For data analysis, independent sample *t*-test was employed to compare two groups. All analyses were conducted using spss24 software.

**Results:**

With respect to students’ skeletal-muscular disorders, findings proved that both genders suffered physical conditions during virtual classes. The research findings showed that the average weekly activity level among women is 634 Met/min with a standard deviation of ±281, and the average weekly activity level among men is 472 Met/min with a standard deviation of ±231. Fat percentage by gender, men’s average fat percentage is 47.21% (S. D ± 4.74) and women’s average fat percentage is 31.55% (S. D ± 4.37). Also, the self-esteem scores of male and female students were obtained 29.72 and 29.43, respectively, and the difference between the two was considered significant (*p* < 0.05). On the other hand, 67% (No. 25) of female students and 32% (No. 12) of male students suffered from high depression. Also, based on students’ skeletal-muscular disorders, findings of our study showed that both genders suffered physical conditions during virtual classes.

**Conclusion:**

This study suggests increasing the level of physical activity to reduce body fat mass, increase mental health and reduce skeletal disorders, which can be properly accomplished through university planning and prioritizing the health of male and female students.

## Introduction

Industrial development, upsetting situation of COVID-19, holding of virtual classes, and life mechanization have created major effects on individuals’ lifestyles and have brought sedentary lifestyles to societies (Nayak et al., 2022). This inactivity in students, as the active class of future society, can cause structural and strategic problems for any country (Yang et al., 2022). Therefore, inactivity should be considered as a serious major problem for the future of the country (Montero-Simó et al., 2022). The role of comprehensive education is in providing education for all humanistic dimensions, which includes not only the intellectual aspect of the individual, but also all psychological and physical aspects (Yuan et al., 2022). This type of comprehensive and inclusive education is beneficial for educational environments such as universities and colleges (Demchenko et al., 2021). Today, in order to better adapt to the surrounding environment, individuals in society need a balance of physical readiness and body composition (Mazza et al., 2020), and if they do not have favorable conditions in terms of physical condition and body composition, they usually become aloof, pessimistic and isolated; in other words, they will not have proper mental balance (Izquierdo, 2005).

Although the measures taken to combat the epidemic have contributed to many people staying at home around the world, this has led people to a sedentary life at home (Chen et al., 2020; Demirci, 2020).

The measures taken to combat the corona disease forced many people around the world to stay at home which led to a sedentary life and weighted gain (obesity; Chen et al., 2020). Obesity is a chronic condition that occurs as a result of intervention in individuals’ genetics or their environment, which is definitely affected by society, culture, psychological, metabolic, biochemical and genetic conditions (Kim et al., 2021). Research findings showed that male students who were living in the city were more likely to be obese (39.4%) than male students in the suburbs (35.5%). Likewise, female students in the city were more exposed to obesity (20.6%) compared to females in the suburbs (19.1%). Findings have shown that male students living in the city are more likely to be obese (39.4%) than male students who live in the suburbs (35.5%). Likewise, 20.6% of female students living in the city were exposed to obesity compared to those female ones (19.1%) living in the suburbs. The increasing prevalence of obesity and overweight among children and adults in the United States of America is a warning for doctors and public health officials (Halpern et al., 2021). In different countries, obesity is probably caused by a decrease in physical activity and an inappropriate way of lifestyle. High levels of health and hygienic indices and physical capabilities reflect the health and potentialities of a society (Wang et al., 2021). It has been well proven that the level of physical activity begins to decrease during adulthood and with age resulting to weight gain, which is associated with weight gain. The evidence that proves the relationship between physical activity in childhood and adolescence and inactivity in individuals in the form of longitudinal studies is rare or does not exist at all. A longitudinal study of 5,700 men and women found a link between childhood activity and obesity in adulthood. It is possible that those who do not engage in sports activities and then become overweight are genetically predisposed to this factor because both physical activity and body size are affected by genetic factors (Chaput et al., 2020). In the last few years, inactivity has become widespread in such a way that since the 1990s, it has been proposed as one of the main factors of death due to cardiovascular diseases (Ralapanawa and Sivakanesan, 2021). Those individuals who have done intense physical activity enjoy better health conditions than those with moderate and light activities (Rosenberger et al., 2019). Studies on the level of inactivity and public health showed that the increase in inactivity is associated with the increase in obesity, as well as decrease in physical activity with a decrease in general health (McCoy and Morgan, 2020). Research related to the level of mobility in different stratum of society has progressed such that it has been shown that the effect of the education level on food intake, obesity and other health risk factors (mobility level) has changed over time (Su et al., 2022). In addition to the fact that regular physical activity leads to an increase in physical readiness, it has been shown that there is a significant relationship between high physical readiness and high self-esteem (Reddy et al., 2021). Some researchers showed that yoga practice and physical exercises strongly influence individuals’ personality, their coping skills and cognitive performance (Pascoe et al., 2021; Farhang et al., 2022; Sinha and Kumari, 2022). Compared to the other groups, Yoga practitioners had higher sattva Guna (balance feature) and preferentially, employed brain regions associated with self-regulation and inhibitory control. Also, other researchers stated that physical activity is essential for children’ current and future health, though most of them do not do 60 min of moderate to intense physical activity daily (Ito et al., 2021; Tandon et al., 2021).

As mentioned, the sedentary life caused by the Corona period leads to obesity, but in addition to the physical effects, it may also affect some psychological factors such as self-esteem (Wang et al., 2020). Studies have indicated that exercising affects self-esteem, and a sense of competence and control. Programs related to physical activity are among the most common ways to increase self-esteem (Mazereel et al., 2021). In addition, compared to complex and heavy activities, simple activities such as aerobic sports have the greatest effect on self-esteem (Ryan, 2008). It has been proved that low self-esteem is related to depression, low mental health and less progress in education (Sánchez-SanSegundo et al., 2022). The impact of physical activity (PA) in reducing the symptoms of depression in children and adolescents has been reported by Dale et al. (2019) through the analysis of 26 articles (Dale et al., 2019). Also, positive effects of PA on the prevention of depression has been reported in older adults (Smaradottir et al., 2020). Despite this, students are prone to depression and examining the role of physical activity on their level of depression is of great importance. a growing body of evidence to suggest that, regular exercise can significantly reduce the risk of depression, anxiety and is considered useful in the prevention of about 25 diseases (Wu et al., 2020). In this regard, evidence from adult studies shows that physical activity is inversely related to depressive symptoms (Kandola et al., 2019). These findings showed Exercise as an intervention for anxiety and depression has been demonstrated in both of the animal studies and human clinical trials.

Beyond the physical conditions of people, COVID-19 is associated with significant mental pressure, which strongly affects mental health (Brooks et al., 2020; Torales et al., 2020). One of the psychological factors that can be related to Corona is self-esteem. Self-esteem is related to dissatisfaction with body image in obese ones who follow weight loss diets (Chang and Kim, 2022). In general, it has been well proven that a decrease in individuals’ self-esteem is related to a decrease in their general health level (Hajek and König, 2019). The relationship between self-esteem and obesity has not been well proven yet (Fields et al., 2021). Although challenges related to self-esteem have significant results on one’s health, due to incomplete results, it is difficult to argue that low self-esteem is a consequence rather than a cause (Bleidorn and Schwaba, 2018). The decrease in muscle volume and general excessive thinness caused by lack of movement endangers health of the body’s skeleton (Jestratijevic et al., 2022). As a result of excessive muscle wasting, the trunk will not be able to perform its functions in maintaining the body and preserving its natural alignment, hence, resulting in bad standing, bad sitting situations, and overall wrong movement habits (Wijngaarde et al., 2020). This makes the spine and chest unable to grow normally and remain in a normal state. In addition to the ugliness and deformity of the body, the unnatural curvature of these organs, causes the blood flow and breathing to not be performed properly and naturally (Hast and Garrison, 2000).

The most important consequences of lack of movement are illness and reduction of muscle volume and strength (Bonnet et al., 2019). The above items are related to each other and there is a direct relationship between the two factors of muscle cross section and the amount of power that a muscle is able to generate. On the other hand, maintaining the skeletal balance of the body is the responsibility of the muscles, especially the amount of strength and power of each muscle. Those individuals who do not have enough physical preparation (readiness), get tired sooner during performance of physical activities. Muscle fatigue in the body will naturally reduce physical ability and reduce the power level that the muscle can represent during working situations (Blocquiaux et al., 2020). In other words, working with tired muscles is the same as working with weak muscles, and the adverse effects that occur as a result of muscle weakness in a person are also like those of working in extreme fatigue conditions. Fatigue and posture may be the cause and effect of each other. In this way, the presence of fatigue caused by other factors such as physical activities in the body can be influential in disrupting the balance of an appropriate posture. On the other hand, lack of having a proper body posture is a reason for causing more fatigue; the more the body size is out of proportion and balance, the more energy is required to keep it straight, because the muscles related to the way the body is positioned to maintain balance have less mechanical merit. On the other hand, they should be involved in activities, which, in turn, will cause body fatigue (Gaudiino and Di Stefano, 2021). If the child’s daily activity is less than normal, the weight of other parts of his/her body will gradually decrease and the weight and volume of the subcutaneous fat tissue will increase, which eventually leads to the child’s obesity. Due to the fact that subcutaneous fat does not accumulate in all parts equally, and in some parts such as around the abdomen, hips, and in general, the middle part of the body is more than the organs (arms and legs), the child’s body position becomes abnormal (Lemaitre et al., 2021).

According to the reports of Center for Disease Control and Prevention in 2018, 29% of school students did not pay attention to physical education classes (Brustio et al., 2018). Meanwhile, another study report stated that students with daily physical activity demonstrate higher academic performance (Páez-Maldonado et al., 2020). Physical activity is a behavior that has many proven health benefits, and it is noteworthy that it is one of the most effective ways to prevent chronic diseases such as coronary heart disease and diabetes (Speelman et al., 2011).

This study aims to determine the frequency and conditions (mental and physical) associated with inactivity in students of Farhangian University during the virtual classes. The main objective will be to measure the level of physical activity and effects of inactivity on mental and physical factors among students studying at Farhangian University of Mazandaran province.

## Research methodology

The method applied in this study is survey type and cross-sectional research design. The statistical population includes all male and female students studying at Farhangian University of Mazandaran province. Students were selected randomly and clustered in such a way that initially the campuses of Mazandaran province were divided based on the city and their population, and students of the cities were randomly selected. The number of samples were selected based on the population; hence, more samples would be allocated to campuses with larger populations; In the following, based on the statistics received from Farhangian University of Mazandaran province, according to the sample size based on Morgan’s Table, 475 students consisting of 214 females and 261 males were randomly selected as the statistical sample of the research. Students with intellectual disabilities, epilepsy, vestibular problem, and hearing or visual impairment were excluded from the study. Ethics approval was obtained from the appropriate institutional ethics review board in Farhangian University. The ethnicity of students was controlled; they were all Iranian. Participants were not told about the purpose of the study. They signed informed consent form and authorized their participation in the study. They were also informed that the data gathered in this study would be kept completely private.

## Research instruments

### The amount of physical activity

After obtaining informed consent, the amount of physical activity was calculated using International Physical Activity Questionnaire (Craig et al., 2003). In this questionnaire, the physical activities performed by the individuals during the last week were asked, and activities performed for more than 10 min were recorded, which included job activities, moving manner, doing household chores, and leisure activities. This questionnaire inquires the amount of intense and moderate physical activity and walking during the last week. According to the scoring protocol of IPAQ questionnaire, the amount of physical activity of a person can be extracted and reported in two ways:

#### The total amount of physical activity of the individual during the last week in terms of MET-minutes/week

MET (Tan et al., 2021) is a unit used to estimate the energy consumption due to physical activity. The value of one MET is approximately equal to the amount of energy consumption of a person at rest. All physical activities can be classified as multiples of the amount of energy consumption in a resting state. In this questionnaire, 3.3 METs for walking, 4 METs are considered for moderate physical activity, and 8 METs for intense physical activity. To calculate the total amount of physical activity in a week, the amount of walking (MET × minutes × day) should be added together with the amount of moderate physical activity (MET × minutes × day) and the amount of intense physical activity (MET × minutes × day) in last week.

#### Classification of individuals’ physical activity in three levels: Low, medium and high

High physical activity means that an individual has intense physical activity at least 3 days a week and a total of at least 1,500 MET-minutes, or that s/he does any combination of intense, moderate, walking activities for seven or more days, with a total of at least 3,000 MET—minutes per week. Moderate physical activity means that an individual has at least 20 min of intense physical activity 3 days a week or more, or that 5 days or more a week has at least 30 min of intense, moderate activity or walking. Low physical activity means that an individual does not report any activity or the reported physical activities do not meet the criteria of high or moderate physical activity (Fogelholm et al., 2006). In the present study, after conducting preliminary studies on the necessity of conducting research on two categories of low and moderate activity intensity, the high physical activity category of the intended samples were disregarded; therefore, the category of high activity level will be excluded from the study, and the current study will be based on the level of activity and percentage of body fat, as well as the level of self-esteem of two classes with a low and moderate level of physical activity.

To determine the validity of the questionnaire, the content validity method was used. Its reliability was measured by the test–retest method, and the correlation coefficient was obtained 0.62 for the awareness and attitude section and 0.74 for the performance section (Moghaddam et al., 2012).

### Fat percentage

Also, the participants’ fat percentage was measured with a Saehan (SH5020) fat meter (caliper) made in England in three points of the body (men: chest, thigh, abdomen) and (women: triceps, upper arm, thigh; Onsori and Galedari, 2015). In order to increase the reliability of the subcutaneous fat measurement process, each part of the body was measured three times with a specific time interval, and all measurements were performed on the right side of the body (Soltani et al., 2018). To determine the fat percentage of the individuals, the measured values were put into Jackson Pollock’s fat measurement formula and the fat percentage was calculated. The standing height of the participants was measured using Height meter model 216 (Seca). For this purpose, the subjects stood such that the rear part of their shoulders touched the height measuring device; They kept their hands next to their body and close to their feet. The weight of each person was measured using a (Seca) model scale.

#### Coopersmith self-esteem questionnaire—short form

Ryden (1978; Morrison et al., 1973) is a 58-item self-report, pencil-paper questionnaire, 8 of which are lie-detectors, and the other 50 items are divided into four subscales of general self-esteem, social self-esteem, family self-esteem, and educational self-esteem. The purpose of this questionnaire is to evaluate students’ self-esteem. This test has different forms. The original test was primarily designed for 8–15-year-old (form A, or school form), but a later revision was designed for subjects over 16 (form C, or adult form). Some items were rewritten to adapt the original form for adults’ use (form C; for instance, children were replaced with individuals, and school with work). There is also a short form of the test (form B, Coopersmith and Brout, 1961) which consists of 25 items and is extracted from the 50-item scale. Coopersmith designed this form as an alternative form for when time is limited. The reliability coefficient of this test is also reported as 0.77. Coopersmith’s self-esteem scale was also standardized in Iran (Narimani and Mousazadeh, 2010).

### Depression scale

Beck depression questionnaire was first developed by Beck et al. (1961) and Jackson-Koku (2016). Beck (1979) made a major revision to cover a wider range of symptoms and provide more consistency with the diagnostic criteria for depressive disorders in Diagnostic and Statistical Manual of Mental Disorders (DSM-IV). Beck’s depression questionnaire is a type of self-assessment test and can be completed in 5 to 10 min. The test consists of a total of 21 items related to different symptoms, in which the participants must answer on a four-point scale from zero to three. These articles cover areas such as sadness, pessimism, feelings of incapability and failure, guilt, sleep disturbances, loss of appetite, self-loathing, etc. Accordingly, 2 items are devoted to emotion, 11 items to cognition, 2 items to overt behaviors, 5 items to physical signs and 1 item to interpersonal semiotics. Thus, this scale determines different degrees of depression from mild to very severe, and its scores range from a minimum of 0 to a maximum of 63. Cronbach’s alpha coefficient of this questionnaire was reported as 0.84 (Farshchi et al., 2018).

### Musculoskeletal disorders

In order to examine skeletal-muscular ailments by the doctor, Nordic questionnaire was employed, which is a standardized questionnaire for examining disorders and disease associated with working and daily affairs (López-Aragón et al., 2017). The reliability of this scale has been reported as 0.73 using Cronbach’s alpha (Namnik et al., 2016).

For data analysis, multivariate analysis of variance (MANCOVA) were conducted on dependent variables with activity group as an independent variable and gender as a covariate. Independent pair sample t-test statistical method was applied to compare two groups. Statistical level of significance for all analyses was set at *p* < 0.05, and effect sizes were calculated as partial ƞ2 (ƞ2p). All analyses were carried out using spss24 software.

## Results

According to the research findings (Demographic characteristics of participants) and the ratio of male and female students in Farhangian University of Mazandaran province, 59% of the participants of this study were male and 41% were female students. The findings of this study showed that 73% of the (No. 348) students had low physical activity and 27% (No. 127) had sufficient physical activity.

Also, among 73% of (No. 348) sedentary students, 40% were women (No. 138) and 60% were men (No. 210). On the other hand, among 27% of students (No. 127) with sufficient mobility, 85% were men (No. 109) and 15% were women (No. 18). Table 1 shows the mean and standard deviation of depression, self-esteem and fat percentage based on gender and activity level.

**Table 1:** Comparison of body fat percentage, self-esteem, and depression of male and female students based on their activity level.

	Sex	Activity	Mean	Number	Standard deviation (SD)	T	sig
Fat percentage	Male students	Active	20.19	71	4.62		
		Inactive	21.54	190	4.43	1.22	0.004
		Total	21.47	261	4.74		
	Female students	Active	30.15	52	4.43	1.45	0.002
		Inactive	31.64	162	4.18		
		Total	31.55	214	4.37		
Self-esteem	Male students	Active	30.82	192	5.47	2.37	0.001
		Inactive	29.45	69	5.16		
		Total	29.72	261	4.37		
	Female students	Active	29.46	164	6.46	2.97	0.004
		Inactive	28.48	50	6.37		
		Total	29.43	214	6.52		
Depression	Male students	Active	20.64	192	6.31	1.86	0.004
		Inactive	27.39	69	6.29		
		Total	25.71	261	6.43		
	Female students	Active	20.52	164	5.67	2.17	0.003
		Inactive	29.61	50	6.44		
		Total	25.65	214	6.30		

**Figure 1:**
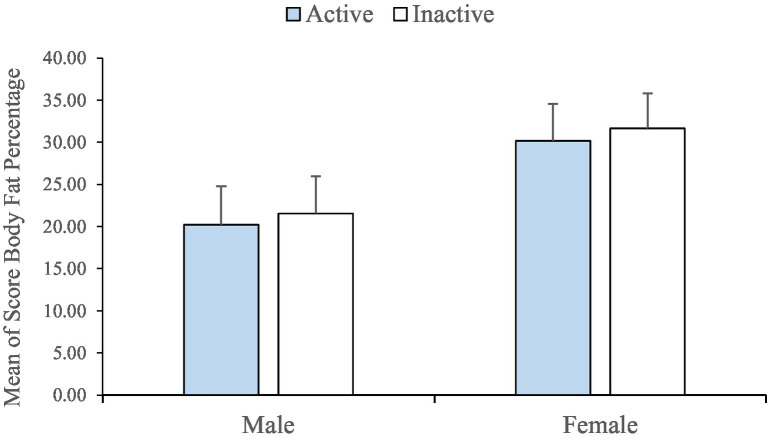
Mean body fat percentage in two active and inactive groups based on gender.

Also, the research findings from examination of pervasiveness of obesity of the students showed that the average fat percentage of the subjects was 25.19 (S. D ± 7.44), which according to gender, the average fat percentage of men was obtained 47.21% (S. D ± 4.74) and the average fat percentage of women was reported 31.55% (S.D. ± 4.37). This difference between men and women is considered significant with *p* < 0.05. Also, the average level of physical activity of all students is 578 Met/min (S.D. ± 284), and the present study showed that the average weekly activity level among women is 634 Met/min with a standard deviation of ±281 and the average weekly activity level among men is 472 Met/min with a standard deviation of ±231 (Table 1; Figure 1).

Also, regarding students’ level of self-esteem, findings proved that 74% of all male and female students (No. 356) had high self-esteem and 26% of them (No. 119) had low self-esteem. Among these 74%, the share of female students is 46% (No. 164) and share of male ones is 54% (No. 192). On the other hand, among those who had low self-esteem, female students accounted for 42% (No. 50) and male ones accounted for 58% (No. 69). However, no significant difference was found between the level of activity and self-esteem.

**Figure 2:**
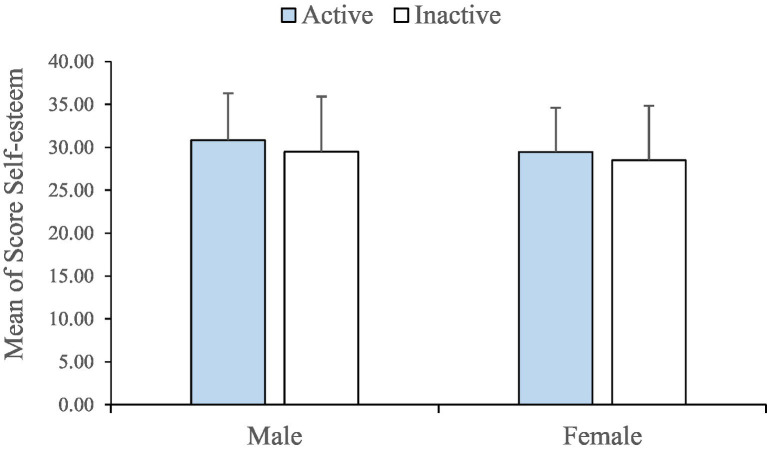
Mean self-esteem scores in two active and inactive groups based on gender.

Based on this, the total self-esteem of the participants was 28.6 (SD ± 5.7). The average score of the students with high self-esteem is 30 and the average score of the students with low self-esteem is 21. Also, the self-esteem scores of male and female students were obtained 29.72 and 29.43, respectively. The difference between the two was considered significant (*p* < 0.05; Figure 2).

**Figure 3:**
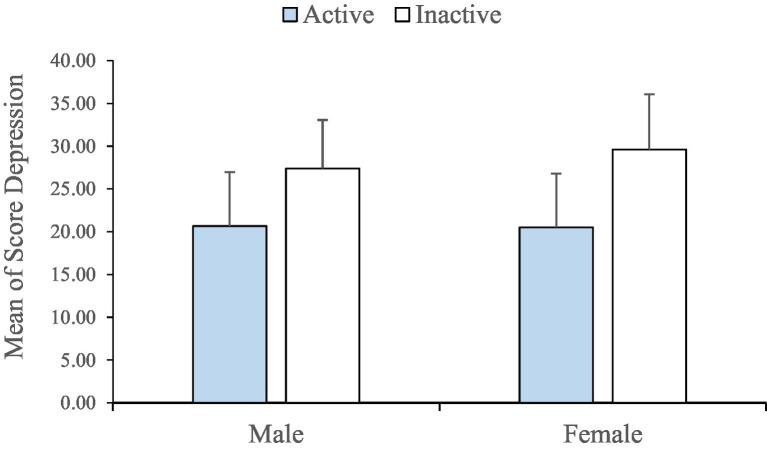
Mean depression scores in two active and inactive groups based on gender.

Also, regarding the level of depression among students, the findings presented that 82% of all male and female students (No. 438) had low depression and 18% of them (No. 37) had high depression. Among these 82%, share of the female students is 45% (No. 203) and share of male ones is 55% (No. 236). On the other hand, female students accounted for 67% (No. 25) and male ones accounted for 32% (No. 12) of high depression. However, no significant difference was found between the level of activity and depression (Figure 3; Table 2).

**Table 2:** Results of MANCOVA in activity and inactivity groups in three variables of self-esteem, fat percentage and depression.

Sources		Sum of squares	Df	Mean squares	F	*P* Value	Eta squares ƞ2
Intercept	Self esteem	332.83	2	166.616	20.763	0.001	0.081
	Body fat	11822.3	2	5911.16	1246.5	0.001	0.842
	Depression	5364.7	2	2682.36	158.38	0.001	0.402
Gender	Self esteem	206.8	1	206.8	25.813	0.001	0.052
	Body fat	11733.03	1	11733.03	2474.31	0.001	0.840
	Depression	98.712	1	98.712	6.04	0.004	0.121
Activity	Self esteem	137.42	1	137.42	17.14	0.001	0.035
	Body fat	175.96	1	175.96	37.107	0.001	0.073
	Depression	5359.02	1	5359.02	316.43	0.001	0.401
	Self esteem	3783.01	472	8.015			
Error	Body fat	2238.19	472	4.742			
	Depression	7993.63	472	16.93			

As illustrated in Table 3, the analysis of MANCOVA with gender as covariate showed that, a significant difference between individuals in two levels of activity (active, inactive) in the variables of self-esteem [*F*(1, 472) = 17.14, *p* = 0.001, ƞp2 = 0.035], body fat percentage [*F*(1, 472) = 37.107, *p* = 0.001, ƞp2 = 0.073] and depression [*F*(1, 472) = 316.43, *p* = 0.001, ƞp2 = 0.401], respectively. The results of independent pair samples *t*-test showed that the active group of active people had better average scores in all three variables of self-esteem, fat percentage and depression, respectively, compared to inactive people, all (*Ps* < 0.05).

**Table 3:** Description and comparison of skeletal and muscular disorders.

Musculoskeletal disorder	Disorder score of active male students	Disorder score of inactive male students	T	Sig	Disorder score of active female students	Disorder score of inactive female students	T	Sig
	Mean ± SD	Mean ± SD			Mean ± SD	Mean ± SD		
All disorders	1.92 ± 2.35	3.92 ± 6.35	2.24	0.003	1.62 ± 3.35	4.92 ± 6.35	3.24	0.043
Total disorders in the last 12 months	1.35 ± 2.48	4.74 ± 5.19	1.98	0.034	1.28 ± 2.97	6.41 ± 3.93	2.62	0.036
Total disorders in the last 7 months	1.56 ± 2.82	5.42 ± 2.32	2.63	0.042	1.67 ± 2.28	7.46 ± 3.14	3.57	0.049
Total disorders leading to prevention of physical activity	2.12 ± 1.47	5.15 ± 2.27	2.86	0.038	1.37 ± 3.83	7.37 ± 2.46	2.64	0.027

## Discussion

The present study aimed to investigate the mental and physical conditions related to inactivity among the students of Farhangian University during the virtual classes due to COVID-19 pandemic. Determining the prevalence and pattern of mental disorders and musculoskeletal pain is the first step in the prevention, diagnosis and treatment of such disorders. This is despite the fact that a targeted and acceptable documented study in this field has not been conducted during COVID-19 era, when students had to use virtual space for classrooms instead of physically attending the class. Therefore, through this research results, appropriate solutions and detailed plans can be taken to alleviate the mental and physical conditions of students.

Results of this study suggests that the prevalence of inactivity among students is high, and about 73% of all individuals did not participate in any of the intense and moderate activities, while in other countries such as Saudi Arabia, the prevalence of physical inactivity includes more than 43% of society (Al-Hazzaa, 2007). In United States, the prevalence of overweight is 36% and obesity is 21% (Davis and Gergen, 1994; Gordon-Larsen, 2001). This amount is reported as 18% (Ramos de Marins, 2001) in Ireland and 33% (McCarthy et al., 2002) in Brazil. Also, the present research results showed that female students had less physical activity (73%) than male ones (51%). Male students demonstrated more physical activity at all levels than female ones. In relation to the number of female students to male ones, the current research results indicated that the activity level of female students was much lower than that of male students, which could be due to the fact that during the Corona period, social restrictions and the closure of sports halls were more for girls. On the other hand, lack of physical activity facilities for both male and female students had a significant impact on their lack of exercise.

Furthermore, researchers have stated that highly-educated individuals have a low level of activity, while other researchers (Lewis, 2005) showed that the amount of physical activity decreases in those with low education. Moreover, based on a study (Hajian-Tilaki and Heidari, 2007), no significant relationship was found between physical activities and education levels, which is consistent with the study of other researchers (Trockel et al., 2000; Wilsgaard et al., 2005). Meanwhile, the average level of physical activity in this study showed that there is a significant difference between the level of physical activity of men and women, i.e., men, participated in this study, have a higher level of physical activity than women. Meanwhile, the average level of physical activity in this study showed that there is a significant difference between the level of physical activity of men and women, i.e., men, participated in this study, have a higher level of physical activity than women. Moreover, during the last three decades, a significant increase in obesity among children and adults has been observed (Carter et al., 2011). This issue has spread to the point that by the increase in individuals’ education level, their body fat percentage increases, too (Marmot, 2003). At the same time, other researchers have reported results contrary to this finding (Morrill et al., 1991) that of the present study, educated ones had a not very high amount of fat mass. The prevalence of obesity in Venezuela is 74% for men and 56% for women (Campos et al., 2003), which is consistent with results of this study regarding the difference between women and men’ level of physical activities. However, this rate in Palestine is 48% for men and 65% for women (Campos et al., 2003), which proves contradictory results with our findings. According to all the findings, the present study showed that the difference in fat mass between sedentary and sufficiently active participants was significant. This means that sedentary ones had a higher fat mass. Another research has shown that the low participation of individuals in educational programs is related to the decrease of their self-esteem, which have a positive correlation (Suss et al., 1996).

This means that by reducing study hours, students’ self-esteem decreases, too; this is in line with results of the present study because the students of Farhangian University had high self-esteem. So, it can be concluded that individuals’ level of self-esteem probably increases by the increase in their education level. Moreover, no significant relationship was observed between the level of activity and high/low self-esteem, which means that both low-activity and sufficient activity groups showed high self-esteem scores; In this regard, other research findings found a significant relationship between self-esteem and obesity (French et al., 1995), which is consistent with the findings of our study; in contrast to these results, another study proved that obese women had lower self-esteem (Pesa et al., 2000). This could be due to the fact that with by the weight increase, the amount of mobility would reduce, and the individual will have fewer social connections and less participation in daily activities, which can possibly reduce his/her self-esteem. In the case of the present study, it can be said that due to the high educational level among the participants and their status in high social and cultural levels, low mobility could not impact their self-esteem. According to the research results (Scherrer and Preckel, 2019), it is stated that self-esteem does not change significantly with changes in the amount of fat mass, in line with the findings of the present study, because there was no statistically significant difference between sedentary and physically active students in their body fat mass. While in an opposite claim (Davis and Gergen, 1994; Guinn et al., 1997; Anderson et al., 2006; Wang et al., 2009), they showed that there is an inverse relationship between individuals’ body weight and self-esteem, i.e., with a decrease in body weight, the amount of self-esteem increases, and with an increase in body weight, self-esteem decreases. On the other hand, other scientists (Childress et al., 1993) claimed that the level of self-esteem among overweight children was significantly lower than their normal counterparts, which is not consistent with the findings of this study, because a significant difference was not observed between the amount of fat mass as well as the activity level with the level of self-esteem, perhaps the reason for this difference can be attributed to the age differences between children and adults.

With respect to the level of depression of Farhangian University students, the findings showed that the level of depression of female students during the COVID-19 pandemic and virtual classes was higher than that of male ones, but this was relatively small, and it can be expected with the increase of psychological and counseling interventions, the case would be reduced. On the other hand, no significant difference was found between the level of activity and depression.

Also, in relation to the degree of the musculoskeletal disorders of the students, findings suggested that among active male students, the highest frequency of pain, discomfort and numbness in last 12 months were related to the wrists and hands (No.17), and in the last 7 days related to back (No. 16), and the most skeletal pain that caused them to stop physical activity in the last 12 months was pain in the knees (No. 21). Also, in sedentary male students, the highest prevalence of pain, discomfort and numbness in the last 12 months was related to the back (No. 42) and in the last 7 days, it was related to the thigh (No. 64), and the most skeletal pain in the last 12 months which made them quit physical activity was related to back pain (No. 74). On the other hand, in female students, the highest prevalence of pain, discomfort and numbness in the past 12 months was related to neck (No. 15) and during the last 7 days, it was related to wrists and hands (No. 22), and the most skeletal pain that caused them to leave physical activity in the last 12 months was related to shoulder pain (No. 31). Also, in sedentary female students, the highest frequency of pain, discomfort and numbness in the last 12 months was related to the back (No. 72), and in the last 7 days, it was related to the shoulders (No. 103), and the most skeletal pain in the last 12 months, which made them quit physical activity was related to back pain (No. 108).

In general, findings of this study are similar to the global statistics of the COVID-19 pandemic and its impact on individuals’ physical and mental factors. It seems that the conditions related to the corona epidemic, as a result, presence of the students in-person, can have negative effects on students. Therefore, it can be concluded that even students and their level of understanding of benefits of physical activity cannot prevent mental and physical problems for them. It is expected that students who use modern scientific resources and are relatively aware of the dangers of obesity and inactivity are not exposed to such injuries, however, in practice, during virtual classes, students of Farhangian University were not sufficiently active; as a result of which physical and mental conditions are observed among them. Also, results related to the degree of psychological factors (self-esteem and depression) showed that in terms of mental health, both female and male students were not in a satisfactory condition due to lack of training and other related factors. It can be concluded that during COVID-19 era, due to health restrictions and government policies regarding quarantine and pandemic, and on the other hand, holding virtual classes and sitting next to communication devices for class, students did not have enough time for exercise. Actually, they did not enjoy physical activity because physical activity was not a priority for students of Farhangian University during the COVID-19.

Therefore, this study suggests increasing the level of physical activity to reduce body fat mass, enhance mental health, and reduce skeletal disorders, which can be properly accomplished through organized university planning and prioritizing health of the male and female students. However, findings of the present study were only limited to the students of Farhangian University in Iran. It is suggested to investigate the effects of inactivity associated with Corona on the physical and mental factors in a wider student’s community of and even students from different nations in future studies.

## Data availability statement

The original contributions presented in the study are included in the article/supplementary material, further inquiries can be directed to the corresponding author.

## Ethics statement

The studies involving human participants were reviewed and approved by Farhangian University. The patients/participants provided their written informed consent to participate in this study.

## Author contributions

MH contributed to the conceptualization, data curation, investigation, methodology, project administration, resources, supervision, validation, visualization, and writing (reviewing and editing) of the study. MP and SNK contributed to the conceptualization, data curation, investigation, methodology, and writing (reviewing and editing) of the study. All authors contributed to the article and approved the submitted version.

## Conflict of interest

The authors declare that the research was conducted in the absence of any commercial or financial relationships that could be construed as a potential conflict of interest.

## Publisher’s note

All claims expressed in this article are solely those of the authors and do not necessarily represent those of their affiliated organizations, or those of the publisher, the editors and the reviewers. Any product that may be evaluated in this article, or claim that may be made by its manufacturer, is not guaranteed or endorsed by the publisher.
